# Lanthanum Inhibits Primary Root Growth by Repressing Auxin Carrier Abundances in *Arabidopsis*

**DOI:** 10.3389/fpls.2017.01661

**Published:** 2017-09-25

**Authors:** Yangyang Liu, Liangliang Sun, Ping Zhang, Jinpeng Wan, Ruling Wang, Jin Xu

**Affiliations:** ^1^Key Laboratory of Tropical Plant Resources and Sustainable Use, Xishuangbanna Tropical Botanical Garden, Chinese Academy of Sciences Mengla, China; ^2^University of Chinese Academy of Sciences Beijing, China

**Keywords:** lanthanum, auxin accumulation, auxin carriers, PINFORMED protein, primary root growth

## Abstract

Lanthanum (La) is one of rare earth elements that was used as a crop growth stimulants; however, high concentration of La markedly inhibited plant growth. Our previous study indicated that, although La induced the expression of auxin biosynthesis-related genes, it markedly repressed primary root (PR) elongation by reducing auxin accumulation in PR tips. In this study, we exhibited that La reduces the abundances of auxin carriers. Treatment with La markedly inhibited the auxins IAA-, 2,4-D-, and NAA-induced elevation of DR5:GUS activity in the roots, suggesting that La inhibited auxin transport through both the influx and efflux transporters. Supplementation with auxin transport inhibitor naphthylphthalamic acid in La-treated seedlings did not further reduce PR growth compared with that of the La treatment alone, further confirmed that auxin transport is involved in La-induced inhibition of PR growth. Analysis of the protein abundances using the transgenic *AUX1-YFP* and *PIN1/2/4/7-GFP* marker lines indicated that La treatment reduced the abundances of all these auxin carriers in the PR tips. La also increased the stabilization of Aux/IAA protein AXR3. Taken together, these results indicated that La treatment inhibits PIN-mediated auxin transport and subsequently impairs auxin distribution and PR growth via reducing auxin carrier abundances.

## Introduction

Lanthanum (La) is an important rare earth element (REE) and has been used as a crop growth stimulants for appropriately 50 years (Hu et al., 2004; Xie et al., 2013; Liu et al., 2016). La affects many aspects of plant growth and development and stress tolerance, including the changes in photosynthesis efficiency, mineral nutrient uptake capacity, antioxidative enzyme activities, and phytohormonal balance (He et al., 2005; Ruíz-Herrera et al., 2012; Wang et al., 2012; Xie et al., 2013; Liu et al., 2016). Although low concentration of La improves crop production, high concentration of La represses crop growth. REE gadolinium (Gd) affects root system architecture (RSA) by inducing a low-phosphorus (P) adaptive responses and the Gd-induced lateral root (LR) formation is inhibited in *tir1afb2afb3* and *arf7arf19* mutants, indicating that Gd modulates RSA by auxin pathway (Ruíz-Herrera et al., 2012). Wang et al. (2014) found that La-activated endocytosis might play a role in La-mediated growth and development in plants. Our previous study found that high concentration of La (≥80 μM) significantly inhibits primary root (PR) growth whereas it markedly induces LR development, and thereby regulates RSA (Liu et al., 2016). However, the physiological and molecular mechanisms underlying La-mediated RSA remain largely unclear.

Auxin plays a central role in modulating root growth and development (Jain et al., 2006; Wang et al., 2009). Auxin signaling is tightly modulated by two protein families, the negative regulators Aux/IAA proteins (Ouellet et al., 2001) and auxin response factors (ARFs) (Ellis et al., 2005), that function directly downstream of the auxin receptor F-box proteins TIR1 and AFBs. The transcripts of *Aux/IAA* genes could be induced by auxin (Muto et al., 2007). The auxin-dependent interaction between auxin receptors TIR1 and AFBs and the Aux/IAA proteins stimulates the degradation of the Aux/IAA proteins, and thereby releasing ARFs proteins and subsequently promoting the expression of auxin-responsive genes (Dezfulian et al., 2016).

Auxin gradients are established and maintained by a network of plasma membrane-localized transporters that facilitate directed auxin influxes and effluxes from individual cells. These transporters include auxin influx carriers of the AUXIN1/LIKE AUX1 (AUX1/LAX) family and efflux carriers of the PINFORMED (PIN) family (Laskowski et al., 2008). Despite the PIN proteins are proposed uniform function in auxin transport, the genetics analysis indicated that different PIN proteins play different roles in developmental processes and abiotic stress responses in plants. For example, PIN1 mediates vascular tissue differentiation, organogenesis (Reinhardt et al., 2003), and auxin redistribution in roots response to glucose and Cu toxicity (Yuan et al., 2013, 2014). PIN2 mediates root gravitropism (Müller et al., 1998) and auxin redistribution in roots response to iron (Fe) stress (Li et al., 2015). PIN4 mediates meristematic activity and auxin redistribution in roots response to biological nitrification inhibitor methyl 3-(4-hydroxyphenyl) propionate (MHPP) (Liu et al., 2016). PIN7 mediates early embryo development (Friml et al., 2003) and auxin redistribution in roots response to manganese (Mn) toxicity (Zhao et al., 2017). Mutations in these transporter genes result in dramatic defects in the RSA and gravitropism. The *pin1pin3pin4pin7* quadruple mutant shows an embryo lethal phenotype. The triple or quadruple mutants show a stronger phenotype than the single mutant, suggested a functional redundancy in PIN proteins (Friml et al., 2003). Yuan and Huang (2016) demonstrated that cadmium (Cd) toxicity reduced auxin level in PR tips by repressing PIN1/3/7 accumulation and increasing IAA17 stabilization. Our previous study indicated that, although La induced the expression of auxin biosynthesis-related genes, it markedly reduced auxin accumulation in PR tips (Liu et al., 2016). However, whether and how La regulates root system growth and development by regulating auxin transport in roots remains unclear. In this study, we investigated the involvement of auxin carriers in La-modulated auxin distribution in PR tips. Our results indicated that La inhibited PR growth by repressing auxin accumulation in PR tips through the reduction of auxin carrier abundances.

## Materials and Methods

### Plant Materials and Growth Conditions

The *Arabidopsis col-0* was used as a wild-type control, and the transgenic lines and mutants used in this study include the following: *DR5:GUS* (Ulmasov et al., 1997), *pAUX1:AUX1-YFP* (Swarup et al., 2004), *pPIN1:PIN1-GFP* (Benkova et al., 2003), *pPIN2:PIN2-GFP* (Blilou et al., 2005), *pPIN4:PIN4-GFP* (Blilou et al., 2005), *pPIN7:PIN7-GFP* (Blilou et al., 2005), *HS:AXR3NT-GUS* (CS9571), *yucca* (Zhao et al., 2001), *pin1* (SALK_047613), *pin4-3* (CS9368), *aux1-7* (CS9583), *pin2* (CS8058), *pin7-2* (CS9366), and *axr3-3* (CS57505). After sterilizing for 5 min with 50% bleach, the seeds were washed five times with sterile water, and then plated onto 1/2 MS medium (Sigma) with 1% agar and 10% sucrose (pH 5.75) and incubated at 4°C for 2 days. The seedlings were grown in a vertical position in a growth chamber (22°C, 16/8 h light/dark cycle). Five-day-old seedlings were transferred onto fresh medium supplemented with 150 μM La(NO_3_)_3_, 0.5 μM IAA, 0.5 μM NAA, 0.5 μM 2.4-D, or 1 μM naphthylphthalamic acid (NPA) for 2 days. For each treatment, at least 20 roots were analyzed, and all experiments were repeated at least three times. The results are presented as the mean ± SD. We used Tukey’s test (*p* < 0.01) for the statistical analyses.

### qRT-PCR Analysis

The root RNA was isolated using RNAiso Plus (TaKaRa) according to the manufacturer’s instructions. Reverse transcription was performed using the PrimeScript^TM^ RT Reagent Kit with gDNA Eraser (TaKaRa). The qRT-PCR was performed with Platinum^®^ SYBR^®^ Green qPCR SuperMix-UDG (Invitrogen). The specific primers for genes are: 5′-TCACGCGGTTACTGTTGAGA-3′ and 5′-TTGGAGTGGTCGAGAAGTGC-3′ for *AUX1*; 5′-ACGGCTGCTGGAACTGCTGC-3′ and 5′-CGTACTGGTTGTCGTTACTATT-3′ for *PIN1*; 5′-TATATTCGGAATGCTGGTTGCTTTG-3′ and 5′-CCATACACCTAAGCCTGACCTGGAA-3′ for *PIN2*; 5′-GTTGTCTCTGATCAACCTCGAAA-3′ and 5′-TATCAAGACCGCCGATATCATC-3′ for *PIN4*; and 5′-CCAAGATTAGTGGAACGCAAC-3′ and 5′-GAAAAGGGTTTTTGGATCCTC-3′ for *PIN7*. All primer pairs were detected only one peak in DNA melting curves, indicating a high specificity of the primers. The qRT-PCR analysis was performed on three biological replicates with three technical repetitions.

### GUS Staining and Measurement of Fluorescence Microscopy

The seedlings with GUS reporter gene were incubated at 37°C in GUS staining solution (0.5 mM potassium ferricyanide, 0.5 mM potassium ferrocyanide, 10 mM EDTA, and 1 mg/ml 5-bromo-chloro-3-indolyl-b-D-glucuronide in 50 mM sodium phosphate buffer, pH 7.0) for 3–5 h (Liu et al., 2016). The GUS staining was quantified in the root tips using ImageJ software. The gray values of GUS staining in the heat-shocked *HS:GUS* and *HS::AXR3NT-GUS* seedlings transferred to 23°C for 1 h without 150 μM La(NO_3_)_3_ treatment was set to 100%, respectively. At least 15 roots were imaged per line for each of three repeats.

The GFP/YFP lines were viewed with a confocal laser scanning microscope (Zeiss, the excitation and emission wavelengths were 488 and 520 nm for GFP and YFP, respectively). For the fluorescence intensity analysis, we selected the full root tips to perform the intensity analysis and obtained the intensity value by confocal microscope. The fluorescence intensity was quantified using ImageJ software. At least 15 roots were imaged per line for each of three repeats.

### Statistical Analysis

Experiments were repeated at least three times, and the results are presented as the means ± SE. The data were analyzed using SPSS (Statistic Package for Social Science) software, and the significance of differences was determined using ANOVA or Student’s *t*-test.

## Results

### Auxin Transport Is Involved in the La-Induced Inhibition of PR Growth

**FIGURE 1:**
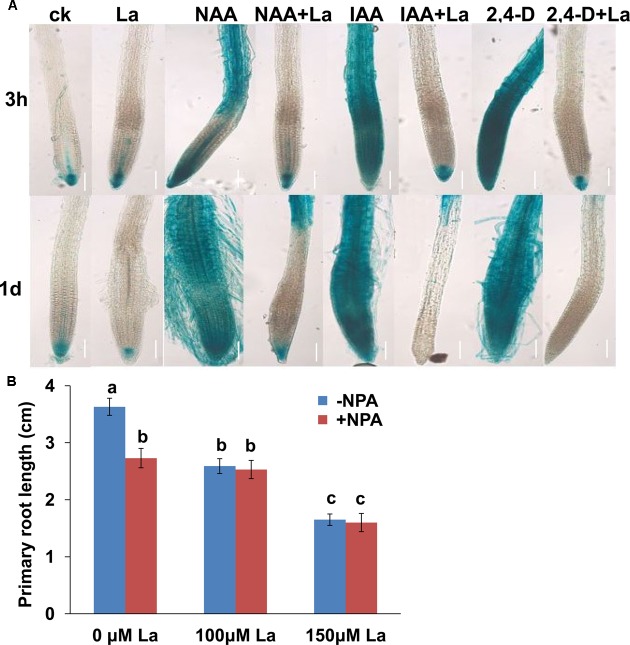
La represses auxin transport in *Arabidopsis* roots. **(A)** Image of GUS staining of 5-day-old *DR5:GUS* exposed to 150 μM La(NO_3_)_3_ and 0.5 μM IAA, 0.5 μM NAA, or 0.5 μM 2.4-D for 3 h and 1 day. Bar, 100 μm. **(B)** Primary root (PR) growth of the wild-type seedlings treated with or without 100 or 150 μM La(NO_3_)_3_ in the presence or absence of 1 μM NPA for 5 days; *n* = 60. The error bars represent the SE. Different letters indicate significantly different values (*p* < 0.05 by Tukey’s test).

Auxin transport is critical for the distribution and accumulation of auxin in root tips, and auxin carriers play a role in this process (Liu et al., 2015). La inhibited auxin accumulation in PR tips (Liu et al., 2016). We thus wondered whether La-mediated auxin accumulation in the PR tips through the modulation of auxin transport. For this purpose, we first explored the potential role of auxin transport in the La-induced repression of auxin distributions in the PR tips using three auxins, IAA, NAA, and 2,4-D. The differences in the transport properties of the auxins IAA, 2,4-D, and NAA have been exploited. NAA enters cells by passive diffusion, whereas IAA and 2,4-D uptake is primarily mediated by an influx carrier (Yamamoto and Yamamoto, 1998). All these auxins induced dramatically elevated DR5-GUS activity in the roots, and treatment with La markedly repressed these processes (**Figure 1A**), suggesting that La inhibited auxin transport through both the influx and efflux transporters. We next examined the effects of NPA, an auxin transport inhibitor, on the La-induced inhibition of PR growth and found that the presence of NPA did not further reduce PR growth compared with that of the La treatment alone (**Figure 1B**).

### La Repressed Auxin Carrier Abundances in *Arabidopsis* Roots

**FIGURE 2:**
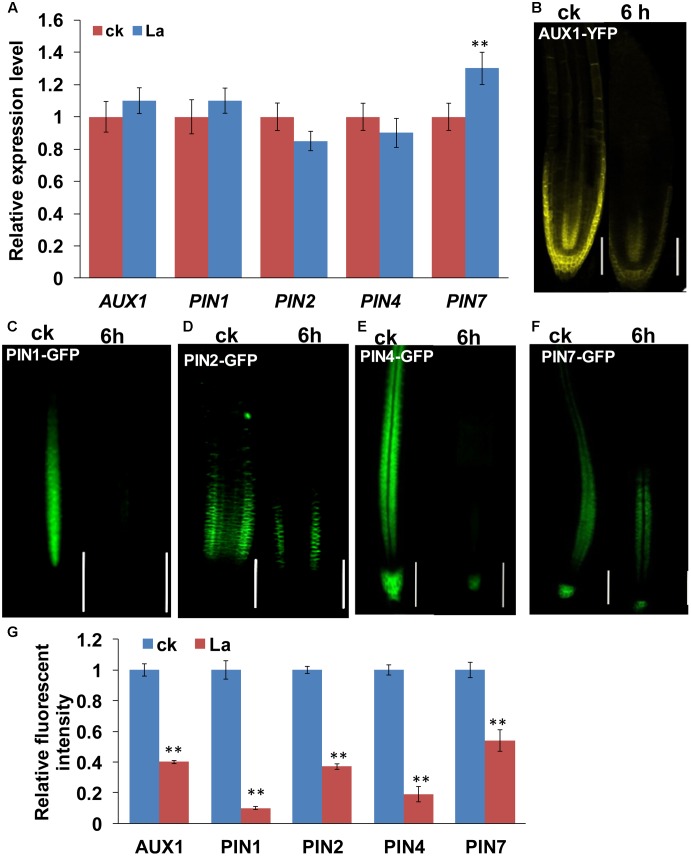
Effects of La on the expression of auxin carriers in *Arabidopsis* roots. **(A)** Real-time quantitative reverse-transcription polymerase chain reaction (qRT-PCR) analysis of the gene expression of auxin carriers in the roots of *col-0* seedlings treated with or without 150 μM La(NO_3_)_3_ for 6 h. The expression levels of the indicated genes in the untreated roots were set to 1. **(B–G)** YFP/GFP fluorescence in the roots of 5-day-old *AUX1-YFP* **(B)**, *PIN1-GFP* **(C)**, *PIN2-GFP* **(D)**, *PIN4-GFP* **(E)**, and *PIN7-GFP* **(F)** seedlings exposed to 150 μM La(NO_3_)_3_ for 3 or 6 h and quantification of the *AUX1-YFP*, *PIN1-GFP*, *PIN2-GFP*, *PIN4-GFP*, and *PIN7-GFP* fluorescence intensity in plants treated as in **(B–F)**. Bar, 100 μm. The error bars represent the SE. Asterisks (^∗∗^) indicate significant differences with respect to the corresponding control (*p* < 0.01 based on Tukey’s test).

**FIGURE 3:**
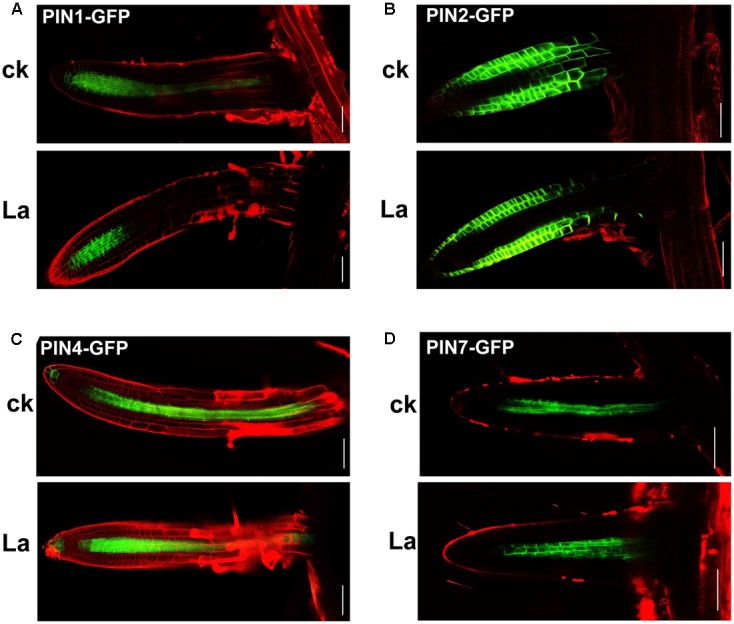
Effects of La on the abundances of PIN1, PIN2, PIN4, and PIN7 in LR. GFP fluorescence in the root tips of the lateral roots (LR) of 5-day-old *PIN1-GFP* **(A)**, *PIN2-GFP* **(B)**, *PIN4-GFP* **(C)**, and *PIN7-GFP* **(D)** seedlings exposed to 150 μM La(NO_3_)_3_ for 6 h. Bar, 100 μm.

**FIGURE 4:**
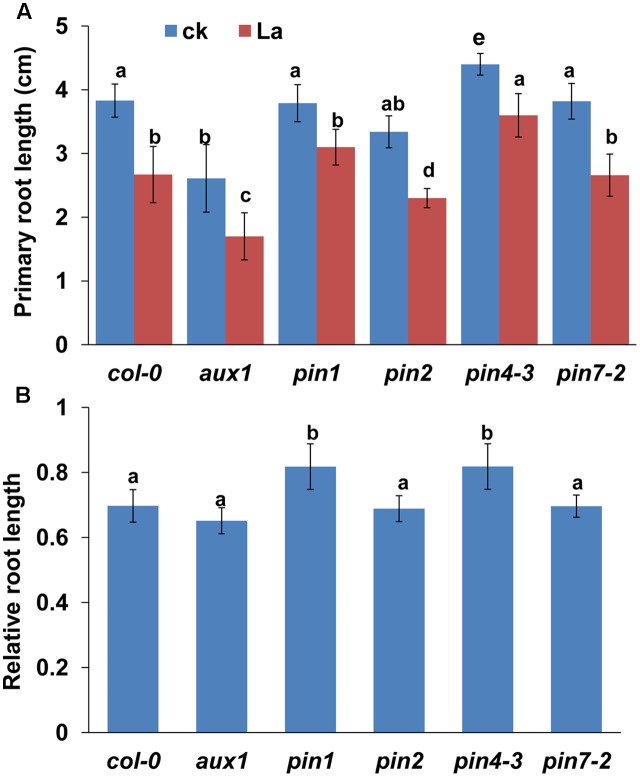
**(A)** PR length of *col-0*, *aux1*, *pin1*, *pin2*, *pin4-3*, and *pin7-2* seedlings treated with or without 150 μM La(NO_3_)_3_ for 5 days. **(B)** The data are presented relative to the La-untreated control values obtained from *col-0*, *aux1*, *pin1*, *pin2*, *pin4-3*, and *pin7-2* seedlings for 5 days; *n* = 60. The error bars represent the SE. Different letters indicate significantly different values (*p* < 0.05 by Tukey’s test).

Auxin transport is mediated by influx carriers of the AUXIN1/LIKE AUX1 (AUX1/LAX) family and efflux carriers of the PIN family (Peret et al., 2012; Yuan and Huang, 2016). Therefore, we examined whether La treatment affects auxin carriers in roots. We first analyzed the gene expression levels of *AUX1* and *PIN1*/2/4/*7* in La-treated roots. As shown in **Figure 2A**, La treatment did not affect the gene expression of *AUX1*, *PIN1*, *PIN2*, and *PIN7*, whereas it increased the level of *PIN7* gene at 6 h of treatment. We then investigated the effects of La on the abundances of AUX1, PIN1, PIN2, PIN4, and PIN7 proteins in the La-treated roots using transgenic lines that express *AUX1: YFP*, *PIN1: GFP*, *PIN2: GFP*, *PIN4: GFP*, and *PIN7: GFP*. The YFP/GFP fluorescence results indicated that the La treatment markedly reduced the abundances of all these auxin carriers in the PR tips, especially PIN1 and PIN4, which La almost fully inhibits their abundances in PR tips (**Figures 2B–G**); however, the expression patterns of these auxin carriers in the LR tips were almost completely unaffected (**Figures 3A–D**). These results indicated that La affects the expression of auxin carriers through post-transcriptional regulation and these analyzed auxin carriers have a role in La-mediated PR growth inhibition. We further explored the roles of these auxin carriers in La-induced PR growth inhibition using the *aux1* and *pin* mutants. The *pin1* and *pin4-3* mutants exposed to La exhibited a smaller reduction in PR growth than the WT seedlings (**Figure 4**).

### Auxin Is Involved in La-Inhibited PR Growth in *Arabidopsis*

**FIGURE 5:**
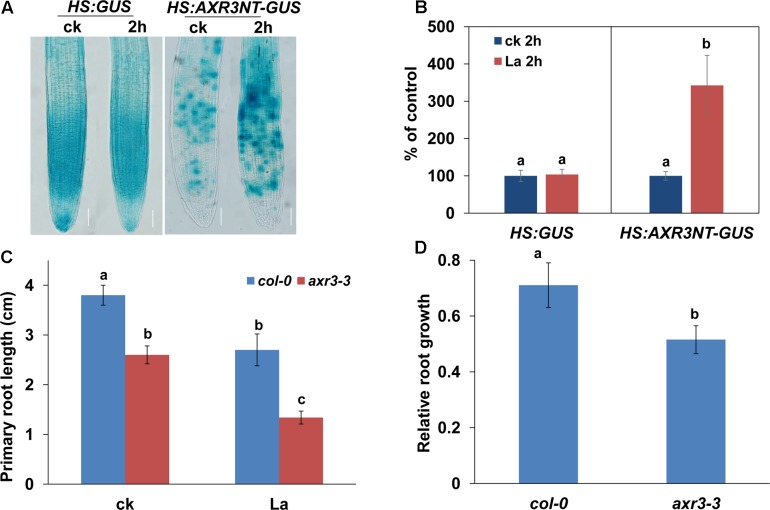
La improves the stabilization of Aux/IAA. **(A)** Image of GUS staining of 5-day-old *HS:GUS* and *HS:AXR3NT-GUS*. The seedlings were heat shocked at 37°C for 2 h and then treated with or without 150 μM La(NO_3_)_3_ for 1 h at 23°C, followed by GUS staining. Bar, 100 μm. **(B)** The relative GUS activity of *HS:GUS* and *HS:AXR3NT-GUS*. The GUS activity in the untreated roots was set to 100. **(C,D)** PR length of *col-0* and *axr3-3* seedlings treated with or without 150 μM La(NO_3_)_3_ for 5 days **(C)**. The data are presented relative to the La-untreated control values obtained from *col-0* and *axr3-3* seedlings for 5 days **(D)**; *n* = 60. The error bars represent the SE. Different letters indicate significantly different values (*p* < 0.05 by Tukey’s test).

Our previous study (Liu et al., 2016) and the present results suggested that the specific repression of the distribution of auxin in PR tips, and not auxin biosynthesis, might be responsible for the inhibition of PR growth by La. To further test the effect of La on the auxin perception in root tips, we used an auxin perceptive *HS:AXR3NT-GUS* reporter line (Gray et al., 2001). After heat shock, the AXR3NT-GUS signal was significantly increased in the PR tips of the La-treated seedlings (**Figures 5A,B**). We then analyzed PR growth in a gain-of-function *axr3-3* mutant (Rouse et al., 1998) in response to La treatment, and our results showed that PR growth in the *axr3-3* mutant was indeed inhibited (**Figures 5C,D**). These data indicated that the reduced auxin accumulation in PR tips improves Aux/IAA stabilization, thereby repressing PR growth.

**FIGURE 6:**
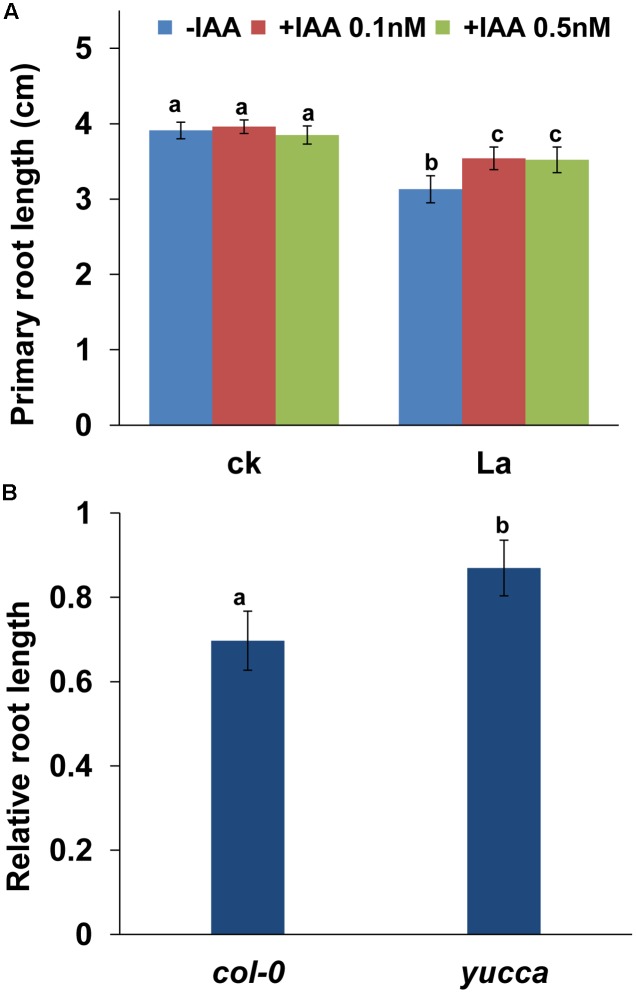
La reduces the accumulation of auxin in root tips, which inhibits PR growth. **(A)** PR growth of the wild-type *col-0* seedlings treated with or without 150 μM La(NO_3_)_3_ plus 0.1 or 0.5 nM IAA for 2 days. **(B)** The relative root length of *col-0* and *yucca* seedlings treated with 150 μM La(NO_3_)_3_ are presented relative to the La-untreated control values obtained from *col-0* and *yucca* seedlings for 5 days; *n* = 60. The error bars represent the SE. Different letters indicate significantly different values (*p* < 0.05 by Tukey’s test).

We then tested the effect of auxin on La-repressed PR growth by applying exogenous auxin and found that supplementation with 0.1 or 0.5 nM IAA alleviated the inhibitory effect of La on PR growth (**Figure 6A**). To further confirm this finding, we also analyzed the PR growth of *yucca*, an auxin over-producing mutant (Zhao et al., 2001), after La exposure. Consistent with the result of pharmacological analysis, the *yucca* mutant exhibited longer PRs than the wild-type (WT) seedlings (**Figure 6B**). These data indicated that La inhibited PR elongation by decreasing the auxin accumulation in the PR tips.

## Discussion

Our previous study indicated that La treatment reduced the IAA contents in PR tips; however, it markedly increased the expression of auxin biosynthesis-related genes in seedlings (Liu et al., 2016). In this study, our results indicated that La inhibits auxin distribution in the PR tips by repressing PIN-mediated auxin transport in PR tips. Several lines of evidence support the conclusion. First, La treatment inhibited IAA-, NAA-, and 2,4-D-induced DR5-GUS activity in the roots, suggesting that disturbing auxin transport by La changes auxin accumulation in PR tips. Second, supplementation with the auxin transport inhibitor NPA did not further increase the La-mediated inhibitory effects on PR growth, suggesting a role of auxin transport in La-mediated PR growth inhibition. Third, an investigation of the abundances of auxin carriers using transgenic lines expressing *AUX1-YFP* and *PIN1/2/4/7-GFP* showed that La repressed the abundances of all these auxin carriers in PR tips, and the *pin1* and *pin4-3* mutants were less sensitive to La-mediated PR growth inhibition, thus confirming that La-repressed auxin transport is involved in the PR growth inhibition response to La. Interestingly, we found that, although La markedly reduced auxin accumulation and the abundances of auxin carriers in PR tips, the auxin accumulation and the abundances of auxin carriers in LR are unaffected. These results indicated that La specifically inhibits auxin transport in PR tips. These data, in combination with our previous study that exhibited the elevated expression of IAA biosynthesis-related genes in seedlings (Liu et al., 2016), might partly explain the enhancement of LR formation in La-treated seedlings. However, the detailed molecular mechanisms involved in La-mediated LR development and how the modulation of the expression of auxin carriers are involved in La-mediated LR formation remain to be further explored.

In this study, we found that the expression of PIN1/2/4/7-GFP and AUX1-YFP was all markedly lower in the La-treated roots than in the control roots, and the *pin1* and *pin4-3* single mutants showed less sensitive to La. The YFP/GFP fluorescence results indicated that PIN1 and PIN4 are almost totally inhibited by La after 6 h of treatment and are more sensitive to La than AUX, PIN2, and PIN7. The result might partly explain the insensitive phenotype of *pin1* and *pin4-3* single mutants to La treatment. Auxin carriers have partially redundant roles in modulating the auxin transport in roots (Blilou et al., 2005). The *aux1* and *pin1* mutant show slightly lower auxin level in root tips (Yuan et al., 2013), whereas *pin2*, *pin4*, and *pin1pin4pin7* triple mutant show higher auxin accumulation in root tips (Friml and Palme, 2002; Blilou et al., 2005; Yuan et al., 2013) than in wild-type *Col-0* seedlings. The increased auxin accumulation in *pin4* root tips could explain the decreased sensitivity of *pin4* mutant to La treatment, however, the decreased auxin accumulation in *pin1* mutant root tips could not explain the decreased sensitivity of *pin1* mutant to La treatment. Similar results were also reported in previous studies (Haga and Sakai, 2012; Liu et al., 2015). Haga and Sakai (2012) found that the pulse-induced positive phototropism is impaired in *pin1*, *pin3*, and *pin7* single mutants and severely in *pin1pin3pin7* triple mutants. Liu et al. (2015) found that the *pin1* single mutant is less sensitive to salt stress, however, the *pin1pin3pin7* triple mutant is less sensitive to both salt stress and exogenous nitric oxide (NO) treatment. Therefore, the reduction of auxin accumulation in PR tips should be a synergistic effect of reduced auxin carrier abundances in La-treated PR tips.

La treatment reduces auxin accumulation in PR tips, and thereby increasing the Aux/IAA stabilization, as indicated by HS:AXR3NT-GUS staining (Katz et al., 2015). Genetics analysis using the gain-of-function *axr3-3* mutant indicated that the increase in AXR3 protein represses auxin signaling, and thereby further aggravating the La-induced PR growth inhibition than the wild-type plants, supported the role of auxin in La-mediated PR growth inhibition. Interestingly, we found that, although La inhibits PIN-mediated auxin transport, exogenous application of auxin effectively alleviates the inhibitory effects of La on PR growth, and genetic analysis supported this result in the auxin over-producing *yucca* mutant. The result could be explained that the local application of auxin in roots or increased auxin accumulation in PR tips could directly modulate root growth in La-treated roots, thereby alleviating La-induced PR growth inhibition. Similar result was also reported in previous studies. Yuan and Huang (2016) found that Cd toxicity markedly inhibits the abundances of PIN1, PIN3, and PIN7 in root tips, and supplementation with exogenous IAA alleviates Cd-repressed PR growth. Taken together, these results indicated that the reduced auxin accumulation in PR tips by repressing the abundances of auxin carriers is responsible for reduced PR growth in La-treated seedlings.

## Author Contributions

JX conceived the study and designed the experiments. YL, PZ, JW, RW, and LS carried out the experiments. JX, YL, PZ, and LS analyzed the data. JX, YL, and LS wrote the manuscript.

## Conflict of Interest Statement

The authors declare that the research was conducted in the absence of any commercial or financial relationships that could be construed as a potential conflict of interest.
